# Analysis of single-cell and spatial transcriptomics in TNBC cell-cell interactions

**DOI:** 10.3389/fimmu.2025.1521388

**Published:** 2025-02-26

**Authors:** Yan Xin, Qiji Ma, Qiang Deng, Tielin Wang, Dongxu Wang, Gang Wang

**Affiliations:** ^1^ Department of Anesthesiology, The Affiliated Hospital of Changchun University of Chinese Medicine, Changchun, China; ^2^ Department of Breast and Thyroid Surgery, The Affliated Hospital to Changchun University of Chinese Medicine, Changchun, China; ^3^ College of Acupuncture, Moxibustion and Tuina, Changchun University of Chinese Medicine, Changchun, China; ^4^ Laboratory Animal Center, College of Animal Science, Jilin University, Changchun, China

**Keywords:** TNBC, single-cell RNA sequencing, cell-cell interactions, spatial transcriptome, immune cells

## Abstract

Triple-negative breast cancer (TNBC) is a highly malignant tumor in women, characterized by high morbidity, mortality, and recurrence rates. Although surgical treatment, radiotherapy, and chemotherapy are the mainstays of current treatment methods, the high heterogeneity of TNBC results in unsatisfactory outcomes with low 5-year survival rates. Rapid advancements in omics technology have propelled the understanding of TNBC molecular biology. The emergence of single-cell RNA sequencing (scRNA-seq) and spatial transcriptomics (ST) has significantly enhanced knowledge of tumor heterogeneity and the distribution, functionality, and intercellular interactions of various cell types within the tumor microenvironment, including tumor cells, T cells, B cells, macrophages, and fibroblasts. The present study provides an overview of the technical characteristics of scRNA-seq and ST, highlighting their applications in exploring TNBC heterogeneity, cell spatial distribution patterns, and intercellular interactions. This review aims to enhance the comprehension of TNBC at the cellular level for the development of effective therapeutic targets.

## 1 Introduction

Breast cancer is a highly prevalent and fatal disease among women, posing a significant threat to their overall well-being. A comparison of the 2012 and 2022 statistics on breast cancer suggests an upward trend in incidence and mortality rates (1–3). Triple-negative breast cancer (TNBC) is a particularly aggressive form of breast cancer, representing around 10–15% of all diagnosed cases (4). It lacks the expression of the estrogen receptor (ER), progesterone receptor (PR), and human epidermal growth factor receptor 2 (HER2) (4). The treatment of TNBC presents significant challenges due to its complex pathogenesis, progressive nature, and high degree of heterogeneity among patients (5). The growth rate of TNBC is rapid, and it exhibits resistance to conventional hormone therapy or HER2-targeted therapy (6). Consequently, the available treatment options are relatively limited and predominantly rely on chemotherapy and radiotherapy. The prognosis for TNBC is generally poor, particularly when it has metastasized to distant sites, with a 5-year survival rate ranging from 4% to 20% (4, 7, 8). TNBC exhibits a pronounced resistance to chemotherapy, and a significant proportion of patients inevitably develop resistance during treatment (9). Immunotherapy shows the potential to address TNBC (10). The immune microenvironment of TNBC, however, remains intricate, and the precise interplay between tumor cells and the immune system has yet to be fully elucidated (11). Therefore, it is paramount to implement novel research techniques to comprehend the immune microenvironment of TNBC and devise more efficacious therapeutic strategies.

With the advancement of sequencing technology, single-cell sequencing and spatial transcriptomics (ST) have demonstrated unique advantages in investigating the tumor immune microenvironment. Single-cell RNA sequencing (scRNA-seq) is a high-throughput technique for analyzing the transcriptome and epigenome at the individual cellular level (12). This methodology enables the acquisition of transcriptomic data at the single-cell level, thereby unveiling the heterogeneity inherent to cells (12). Single-cell sequencing involves isolating and sequencing a single cell’s genome, transcriptome, or epigenetic profile. This method enables the accurate identification of distinct cell subsets and provides insights into their gene expression patterns and functional characteristics (13). Applying scRNA-seq to TNBC tumor samples enables the identification and classification of diverse cellular subtypes, encompassing tumor stem cells, epithelial cells, and immune cells (14). The interaction between different cell subtypes of TNBC can also be investigated using scRNA-seq, revealing that endothelial cells within TNBC tumors play a crucial role in maintaining tumor stem cells through the NOTCH4 signaling pathway (15). Additionally, scRNA-seq has the potential to investigate the evolutionary aspects of tumor cells, including their development and metastasis. Examining the transcriptome of metastatic TNBC cells and primary tumor cells enables the discovery of crucial biomarkers and signaling pathways linked to the process of metastasis (16, 17). The limitations of scRNA-seq in capturing spatial information on cellular localization within tissues hinder the comprehensive comprehension of cell-cell interactions and the tissue microenvironment. To address this issue, the advancement of spatial transcriptomics has been advocated.

ST is an impartial, high-capacity technique for identifying gene locations within a spatial context (18). The primary characteristic of ST involves the fusion of transcriptomics and high-resolution tissue imaging, facilitating the correlation between gene expression patterns and the spatial arrangement within an organ or tissue (19). By preserving spatial information on tissue sections, ST enables the visualization of cell type distribution and their interactions within the tumor microenvironment, thereby providing insights into tumor heterogeneity and evolution (20, 21). The spatial arrangement of cells in TNBC holds significant prognostic implications. A previous study demonstrated a strong correlation between patient prognosis and the presence and spatial positioning of lymphocytes, such as CD8^+^ T cells (22). ST facilitates the comprehension of immune cells distribution within the tumor microenvironment of TNBC, thereby improving the knowledge of cellular interactions associated with tumor growth and metastasis (23). For example, tumor cells can affect immune cell function through the release of cytokines, which facilitate the growth and metastasis of tumor tissue (24, 25). ST can provide valuable insights into the dynamic remodeling process of the tumor microenvironment. The essential role of the reciprocal communication between tumor cells and stromal cells in regulating the tumor microenvironment, leading to enhanced tumor advancement and metastasis, has been well established (26).

ScRNA-seq and ST techniques offer novel insights into TNBC. Tumor cell composition, the tumor microenvironment, and the intricate interactions between tumor cells and immune cells have been comprehensively analyzed using scRNA-seq and ST techniques to understand tumor cell heterogeneity and the immune response. This information is beneficial for determining therapeutic targets and advancing personalized treatment for TNBC. This review covers tumor heterogeneity, the immune microenvironment, and cell-cell interactions as analyzed in TNBC using scRNA-seq and ST.

## 2 Characteristics of scRNA-seq and ST

Due to the intricate and heterogeneous nature of the tumor microenvironment, bioinformatics analysis is insufficient for understanding stromal cells, immune cells, and tumor cells within this environment. This limitation has stimulated the advancement of scRNA-seq and ST.

### 2.1 ScRNA-seq

**Figure 1:**
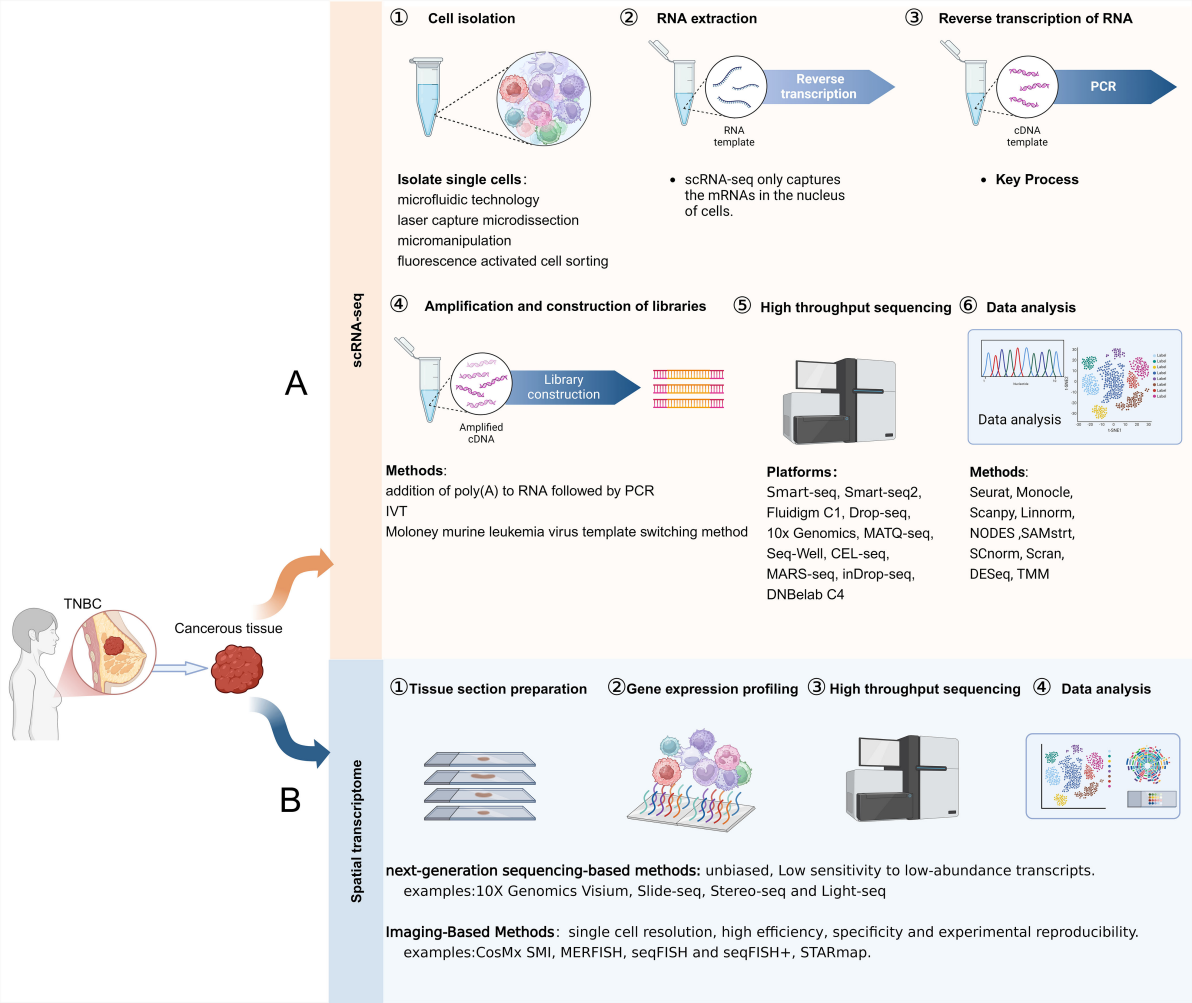
Steps of ScRNA-seq and ST. **(A)** The steps involved in scRNA-seq. **(B)** The steps involved in ST.

ScRNA-seq is a high-throughput technique that enables transcriptome analysis at the single-cell level. Technology involves isolating a single cell, extracting its RNA, and sequencing it to obtain cellular gene expression or genomic information (27). This method facilitates the recognition of various cellular categories and functional conditions, as well as intercellular heterogeneity (28). Unlike traditional RNA-seq, which only provides information on a sample’s overall gene expression level, scRNA-seq can reveal cell-to-cell heterogeneity (29, 30). There are two primary methods for categorizing scRNA-seq: sequencing of full-length transcripts and sequencing of the 3’/5’-end of transcripts (31). The process of scRNA-seq includes the isolation of cells, extraction and reverse transcription of RNA, amplification and construction of libraries, high-throughput sequencing, as well as data analysis (**Figure 1A**).

### 2.2 ST

The field of ST integrates transcriptomics and spatial biology to visualize the cellular distribution within the tumor microenvironment by capturing gene expression information from tissue slices (32). ST complements scRNA-seq by providing crucial spatial information, enabling the identification of cell type distribution patterns, cellular localization in tissues, and intercellular relationships (33). Based on the data acquisition method, ST is divided into two categories: imaging-based methods (*in situ* hybridization and *in situ* sequencing) and next-generation sequencing (NGS)-based methods (microanatomical specimens and NGS with spatial barcodes) (34, 35). The steps involved in obtaining spatially resolved transcriptome information are tissue section preparation, gene expression profiling, high-throughput sequencing, and data analysis (**Figure 1B**). ST has been employed to analyze tumor heterogeneity, explore the relationship between immune cells and tumor cells in the TNBC tumor microenvironment, and investigate the spatial distribution patterns of biomarkers in TNBC (35, 36).

ScRNA-seq and ST comprise comprehensive and in-depth analysis tools for TNBC, enabling the acquisition of detailed and comprehensive gene expression information. They facilitate the analysis of TNBC heterogeneity and the immune microenvironment, resulting in the identification of new therapeutic targets and markers. Moreover, these technologies contribute to advancing personalized therapy and precision medicine.

## 3 Analysis of cell subsets in TNBC by scRNA-seq

**Figure 2:**
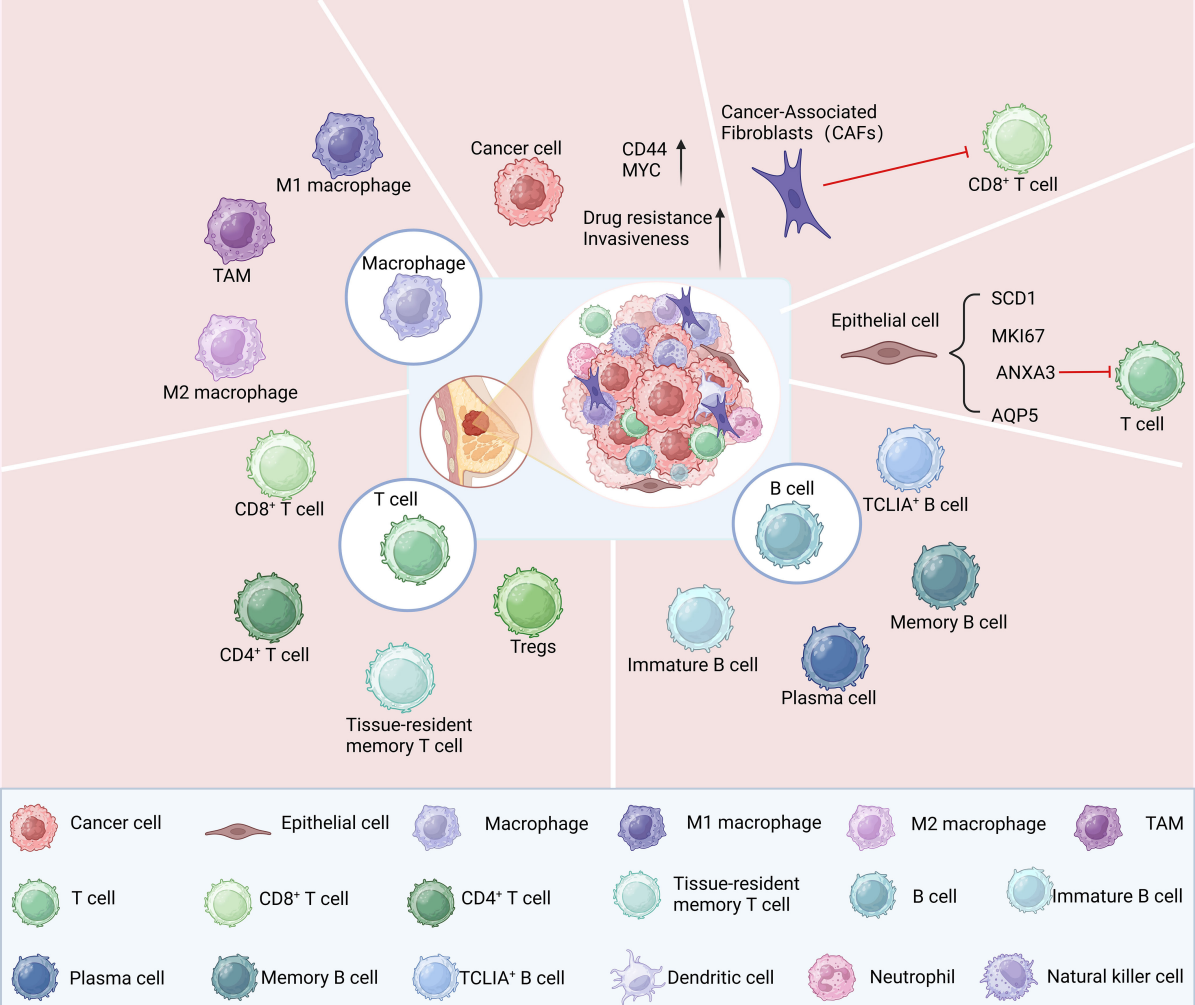
Cell subsets within TNBC identified through scRNA-seq analysis.

The aggressive cancer subtype known as TNBC exhibits a notable degree of cellular diversity. Revealing gene expression traits becomes feasible with the utilization of scRNA-seq in distinct cell subsets and facilitates the detection of intercellular heterogeneity in single-cell resolution (37). Applying scRNA-seq to TNBC cells facilitates the discovery and analysis of unique cell subsets, providing valuable insights for tumor prognosis and treatment (**Figure 2**).

### 3.1 Tumor cells and epithelial cells

ScRNA-seq analysis has unveiled the presence of diverse cellular populations in TNBC samples, encompassing cancer-associated fibroblasts, epithelial cells, immune cells, and tumor cells (38). The transcriptome characteristics of these cells exhibited notable disparities, which accurately reflected the tumor’s heterogeneity (39). The presence of tumor stem cell populations in TNBC was identified through scRNA-seq, revealing their pronounced expression of cancer stem cell (CSC) characteristics, such as elevated levels of CD44 and MYC gene expression (40, 41), that confer the ability to survive and proliferate within tumor tissue. Additionally, scRNA-seq revealed that the elevated expression of these genes conferred heightened resistance and aggressiveness upon TNBC (42). scRNA-seq analysis revealed that four distinct subsets of epithelial cells, namely SCD1, MKI67, ANXA3, and AQP5, had significant pathological implications for TNBC. Furthermore, cell-cell interaction analysis demonstrated that ANXA3-expressing epithelial cells exerted suppressive effects on T cell function through the BTLA-TNFRSF14 pathway (43).

### 3.2 T lymphocytes

The functions of immune cell populations in TNBC were determined by conducting scRNA-seq analysis of 9683 immune cells that had infiltrated the tumor (44). The proportion of CD8^+^ T cells in TNBC has been consistently observed to be consistently high across various studies; however, their functionality may be impaired, leading to an exhausted state marked by the increase in inhibitory markers like PD-1 (45–47). The T5 cluster (CD8^+^ T Cells) in TNBC indicates the “pre-depleted” state of T cells, while the enrichment of CXCL8-CD8 naive T cells (T2) could potentially facilitate the swift advancement and spread of TNBC (44). The infiltration characteristics of tumor-infiltrating lymphocytes (TILs) are significantly pronounced in TNBC. A strong association has been established between the presence of TILs and the prognosis of patients. Specifically, elevated levels of CD8^+^ T cell infiltration serve as a reliable predictor for improved survival rates and a reduced risk of relapse (48). Tissue-resident memory T cells (TRMs) are crucial in the tumor microenvironment, promptly responding to tumor antigens and promoting anti-tumor immune responses. Studies have demonstrated a positive correlation between the abundance of TRMs and survival rates in patients with early-stage TNBC, suggesting a more favorable prognosis than CD8 expression alone (49, 50). Crucial for immune tolerance maintenance and suppression of excessive immune responses, regulatory T cells (Tregs) play a vital role. However, the proportion of Tregs increases in TNBC, resulting in immunosuppression and a poor prognosis (51).

### 3.3 B lymphocytes

The abundance and distribution of B cell subsets in tumors significantly impact the occurrence, progression, and prognosis of TNBC. A significantly elevated presence of B cells within TNBC tissues compared to normal tissues has been noted (46). This is closely related to the alteration of the tumor microenvironment, whereby cytokines secreted by the tumor facilitate the recruitment and proliferation of B cells (46). Additionally, a significant disparity was observed in the composition of B cell subsets. Research indicates that approximately 10% of TIL-Bs comprise immature B cells, while plasma cells account for around 20%. The majority, constituting about 80%, are memory B cells, revealing their primary function in the immune defense against TNBC (52). However, Breg cells were not observed in either TNBC patients or mouse models (53). TNBC-infiltrating memory B cells exhibit heightened clonality and undergo extensive IGH class switch recombination (CSR) and somatic hypermutation (SHM), potentially occurring within the tumor microenvironment while recognizing tumor antigens (53). The transcription profile of the TIL-B gene may be associated with a higher survival rate among patients with TNBC and offer a more favorable prognosis compared to the classical B-cell marker (CD20) (53). The presence of TCL1A^+^ B cells positively correlated with the anti-midstream immune microenvironment and overall survival (OS) (54). These findings imply that the combination of TCLIA^+^ B cells is crucial in the management and prognosis of TNBC (54). The B cells present in TNBC tumors can be categorized into two primary subsets: CD19^+^ B cells and plasma cells. The relative proportion and functionality of these distinct B cell subsets significantly impact the immune microenvironment within tumors, as well as patients’ prognostic outcomes. Other studies have demonstrated that B cells have a propensity to undergo differentiation into plasma cells, a process facilitated by the interaction between B cells and T cells. This phenomenon may explain the T-cell-mediated immunosuppression observed in TNBC (46). A report indicated that elevated levels of CD19^+^ B cells are correlated with improved clinical outcomes, whereas an abundance of plasma cells could potentially contribute to the development of tumor drug resistance and a less favorable prognosis (55). This is related to the functions of CD19^+^ B cells and plasma cells. CD19^+^ B cells can regulate the tumor microenvironment by secreting a variety of cytokines and chemokines, such as IL-10, IL-6 and TNF-α, and produce specific antibodies against tumor antigens to inhibit tumor growth (52, 56–58). In addition, it promotes the activation and proliferation of CD4^+^ T cells through interaction with them (59), and provides tumor-specific antigens to CD8^+^ T cells to enhance their cytotoxicity (60). Study has shown that plasma cells can release cytokines to suppress the responses of T cells, thereby reducing the immune responses against tumor cells (61). The specific antibodies produced by plasma cells can neutralize chemotherapy drugs or protect tumor cells from the effects of drugs, thus leading to drug resistance (62).

### 3.4 Macrophages

Macrophage subsets within TNBC tumors frequently co-express markers associated with both M1 and M2 phenotypes, implying that macrophages contribute to anti-tumor responses and facilitate the progression of tumors and their spread in the tumor microenvironment (55). Normal human breast tissue predominantly contains CD206^+^ macrophages, while an increase in the proportion of CD206^−^ macrophages was observed in tumor tissue (63). The presence of CD206 is commonly associated with M2-type macrophages, suggesting that these may dominate the microenvironment of TNBC (63). Gene expression and cluster analysis of untreated TNBC tumors revealed a significant upregulation in the gene expression of M2-type macrophages, which may facilitate the development of tumors by releasing substances that hinder immune response (64). Additionally, specific subsets of tumor-associated macrophages (TAMs) exist within TNBC tumors, correlated with T cell infiltration and immunosuppression, emphasizing the crucial function of macrophages in controlling the immune microenvironment of tumors (46, 65). Therefore, in the immune evasion of TNBC tumor cells, TAMs can inhibit T cell function and weakening the immune response, facilitating a conducive microenvironment for tumor growth (66, 67).

In addition, a positive relationship was found between the presence of macrophages and the survival rate in patients with TNBC. Subsets of CD206^+^ macrophages expressing SERPINH1 and collagen 1 or MORC4 were associated with improved survival, while CD206^−^ macrophages were linked to a poorer prognosis (63). According to a recent investigation, CD163^+^ macrophages within the tumor microenvironment of TNBC are correlated with improved survival outcomes. These macrophages exhibit anti-inflammatory characteristics and may impede tumor progression by modulating the tumor’s immune environment. Transcriptome analysis revealed a significant correlation between a high density of CD163^+^ macrophages and improved OS, as well as TNBC-specific survival (BCSS) in patients, offering fresh perspectives on the involvement of macrophages in TNBC (68).

The TNBC microenvironment is influenced by the intricate interplay between tumor cells and immune cell subsets. ScRNA-seq analysis offers valuable information on the diversity and functional properties exhibited by these cellular populations, thereby guiding treatment strategies. Integrating scRNA-seq with multi-omics analysis can facilitate the understanding of the intricate cell-to-cell interactions and tumor microenvironment in TNBC, thereby enabling personalized treatment strategies for TNBC patients in the future. scRNA-seq has significantly enhanced the comprehension of tumor cell subsets, the immune microenvironment, and tumor heterogeneity. Compared to scRNA-seq, ST technology can precisely localize gene expression in space (69).

## 4 Immune microenvironments of TNBC

Comprehending the intricate tumor microenvironment holds the key to deciphering the pathogenesis of TNBC. The recent progress in scRNA-seq and ST has opened novel and unprecedented avenues for exploring the heterogeneity and spatial organization of cells within tumors. These techniques are instrumental in dissecting the TNBC tumor microenvironment and in-depth analyzing the interactions among cells as well as their underlying mechanisms.

### 4.1 Spatial distribution characteristics of immune cells in TNBC

The microenvironment of TNBC comprises tumor cells and immune cells. The intricate interactions between these cellular components pose significant challenges in TNBC treatment, emphasizing the utmost importance of investigating their interplay (23). ST technology, which integrates the benefits of transcriptomics and spatial biology, enables a comprehensive examination of cell-to-cell interactions while preserving gene information at various resolutions, including multicellular, single-cell, and even subcellular. This is essential for comprehending the intricate microenvironment of TNBC and inter-cellular interplay (70). Studies have revealed variations in the composition and quantity of immune cells among different TNBC subtypes. For instance, the basal-like subtype is typically characterized by heightened levels of CD8^+^ T cell infiltration and macrophages, suggesting a reliance on cell-mediated immune responses. Conversely, the immunoactive subtype may exhibit a greater abundance of B cells and activated T cell infiltration (71, 72). The spatial distribution of immune cells may also vary between subtypes (73). Additionally, different tumor regions within the same subtype can exhibit distinct distribution patterns.

The dispersion of different types of immune cells, encompassing B cells, T cells, and macrophages, within tumor tissues can influence tumor growth and metastasis. Research has demonstrated substantial variability in tumor cell density; however, immune infiltration exhibits coordination, with significant positive correlations observed between the density of immune cells and B cells alongside negative correlations with macrophages (74). Tumor cells exhibited significant co-localization with T cells, particularly CD8^+^ T cells (75). The presence of CD8^+^ T cells in long-term survivors suggests a correlation between CD8^+^ T cell activity at the interface of cellular neighborhoods and antitumor immune responses (74). The proximity of Treg and Tex cells to tumor cells was significantly enhanced in long-term survivors (74). T cells in contact with cancer cells exhibit enhanced proliferative activity and express elevated levels of activation markers. The spatial composition of TNBC’s cellular structure is associated with cell-cell interactions, closely linked to the prognosis of patients undergoing immunotherapy (76). The number and distribution of infiltrating immune cells are closely associated with the tumor’s biological behavior in TNBC.

ScRNA-seq can provide gene expression information at the cell level. However, it has obvious limitations in determining the spatial location of cells and the interactions between cells in different regions. In contrast, ST can obtain gene expression data while retaining the spatial location information of cells, which offers a crucial advantage for deeply exploring the complex microenvironment within tumors. There are distinct disparities in the spatial arrangement of immune cells between the central region and periphery of TNBC tumors. Specific subsets of T cells, particularly cytotoxic T cells (CD8^+^ T cells), frequently accumulate in densely populated regions at the tumor margin, indicating a favorable prognosis (23). The analysis of tumor core and marginal regions using ST revealed that the tumor core regions typically exhibit an immunosuppressive microenvironment, while the marginal regions tend to be enriched with active immune cells (77). This precise analysis of the specific expression patterns in different regions of the tumor is beyond the reach of scRNA-seq. Through the ST technology, the differences in the gene expression profiles of immune cells in the tumor core and the marginal regions can be intuitively observed, as well as how these differences are closely related to the biological behavior and clinical outcomes of the tumor. The integration of scRNA-seq and ST data enables the identification of immune cell types and their functional states within the tumor microenvironment, facilitating a more comprehensive exploration of the intricate interplay between tumors and the immune system (23, 73).

### 4.2 Cell-cell interactions in TNBC

**Figure 3:**
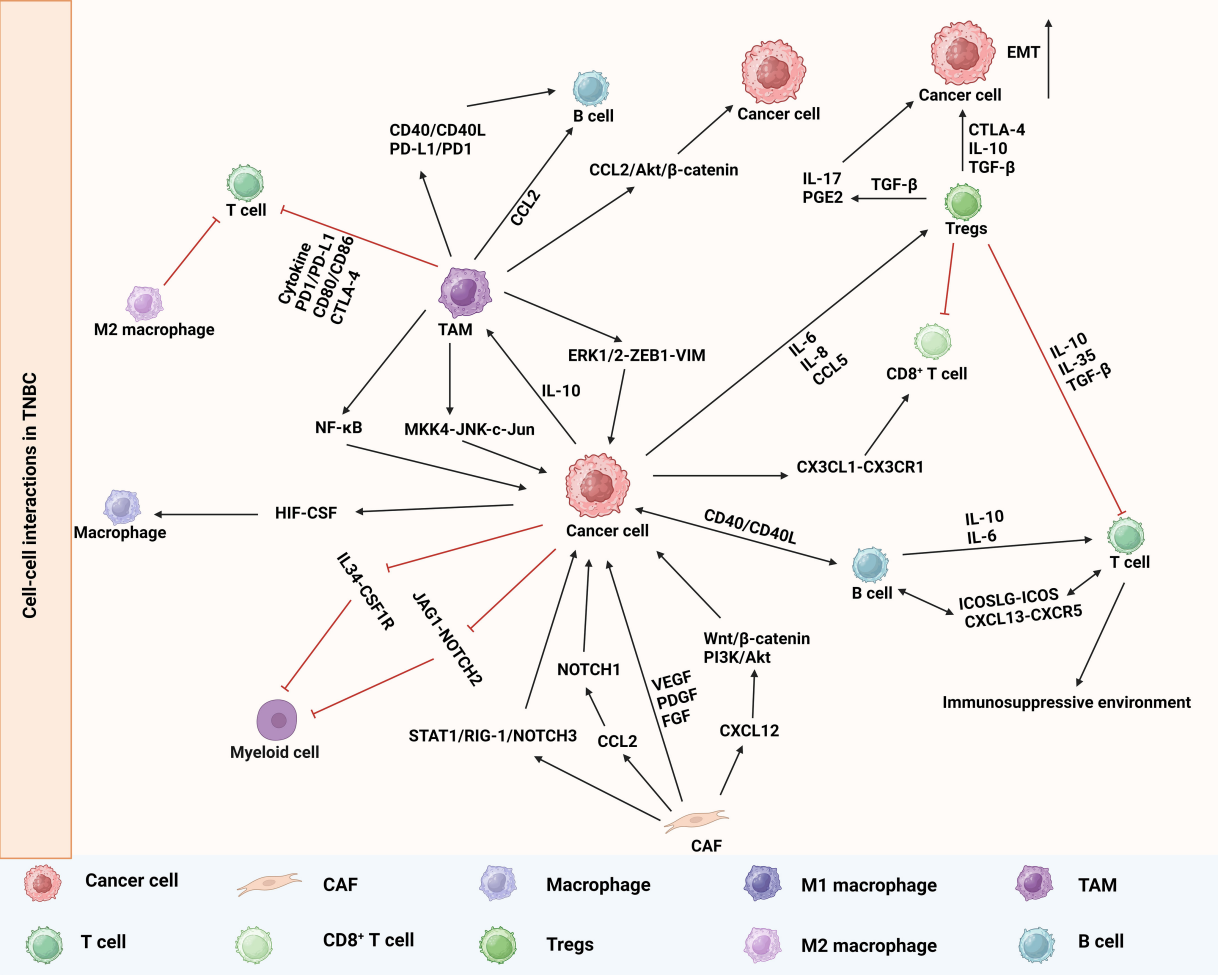
Cell-cell interactions in TNBC recognized by scRNA-seq and ST.

The growth and metastasis of TNBC are heavily influenced by cell-cell interactions (78). ScRNA-seq and ST enable the analysis of immune cell and stromal cell interactions in the tumor microenvironment, as well as their interactions with tumor cells (**Figure 3**). These intricate cellular communications facilitate the survival and proliferation of CSCs, thereby exerting a profound impact on tumor progression (79, 80).

#### 4.2.1 TAM-cell interactions

The diversity and functionality of TAMs are intricately linked to their specific localization within tumors and interactions with neighboring cells. The integration of scRNA-seq and ST in certain studies revealed the involvement of cell-to-cell interactions in the initial dissemination of breast cancer, as well as the activation of the macrophage migration inhibitory factor pathway between disseminated tumor cells and immune cells (81). The promotion of tumor metastasis by TAMs is attributed to their ability to modify the tumor microenvironment (82, 83). Abundant evidence indicates a strong correlation between the extent of TAM infiltration and the metastatic potential of tumors (84). A study demonstrated that TNBC tumor cells possess a heightened capacity to induce M2 macrophage differentiation. TAMs activate the downstream ERK1/2-ZEB1-VIM pathway through IL1α binding to IL1R1, thereby promoting TNBC cancer cell metastasis. Additionally, TAMs can stimulate IRAK4 to activate the MKK4-JNK-c-Jun and NF-κB pathways, consequently enhancing the expression of IL1α, IL1β, and IL-8 in TNBC cells (85). ST data have revealed specific spatial associations between TAMs and tumor-associated fibroblasts, which are sustained through cytokine-mediated interactions (86). TAMs enhance the functionality of CAFs through cytokine secretion, thereby promoting tumor growth and facilitating tumor progression. Importantly, TAM-induced alterations in the transcriptome of breast cancer cells are significant and comprehensive, encompassing transcription regulation, translation control, cellular transport mechanisms, the dysregulation of cell cycle processes, and immunosuppressive effects (87). These findings imply that TAMs could potentially serve as targets for the treatment of TNBC.

The expression of M2-type TAMs is significantly upregulated in TNBC, and scRNA-seq revealed the abnormal expression of the characteristic genes MS4A6A and PLAUR in TNBC tumor tissue macrophages (88). M2-type TAMs exert an immunosuppressive effect, which can impede the functionality of T cells and impact the anti-tumor immune response (89, 90). The inhibitory effect of TAMs can be exerted through the secretion of cytokines, such as IL-10 and TGF-β, which form a complex network to suppress T cell activity and impair T cells’ anti-tumor capabilities (91, 92). The release of IL-10 by TAMs additionally hinders the antigen presentation capability of DCs, thereby impeding tumor immunity (93). The spatial distribution of cells was compared between treated and untreated TNBC patients. In treated patients, the distance between APOE macrophages and CD8^+^ Tex cells was found to be greater than in untreated ones, while the distance between APOE macrophages and CD8^+^ cytotoxic T cells was reduced (94). These findings suggest that the interaction between macrophages and T cells influences immunotherapy (94). The association between TAMs and T cells can also be established through receptor-ligand interactions. For instance, TAMs express PD-L1 and engage with PD-1 on cells to suppress T cell activity and facilitate immune evasion by tumors (95). Additionally, CD80/CD86 and CTLA-4 can regulate T cell function and inhibit excessive activation (96–98).

#### 4.2.2 T lymphocyte-cell interactions

The interaction between T lymphocytes and various cells within the tumor is crucial in shaping the immune microenvironment of TNBC. Treg cells play a significant role in tumor immune evasion in TNBC. Treg cells can suppress effector T cell responses against both foreign antigens and autoantigens, including tumors (99). This leads to the development of an immunosuppressive microenvironment within the tumor, facilitating immune evasion and promoting tumor growth. The tumor microenvironment of TNBC was found to exhibit a high degree of immunosuppression, as evidenced by scRNA-seq analysis revealing an increased infiltration of Treg cells in TNBC, which subsequently hindered the cytotoxic function of CD8^+^ T cells and thereby facilitated cancer progression (100). Spatial analysis revealed the proximity of Treg cells and effector T cells, forming immune hotspots, wherein the suppressive effect of Treg cells dampened the immune response in these regions (101, 102). These findings also further support the impact of Treg infiltration-induced immunosuppression on the TNBC microenvironment (103). Cellular transcriptome analysis further revealed elevated levels of Tregs (CD4_c06_Treg_FOXP3) and exhausted CD8^+^ T cells (CD8_C10_ence-based sted_HAVCR2). Transcriptome analysis of Tregs revealed the enrichment of the co-stimulatory genes LGALS9, TNFRSF4, CD27, and CD28, as well as the immunosuppressive genes IL2RA, IL2RB, IL2RG, ENTPD1, and LAG3. These findings suggest that Tregs in TNBC have a pronounced immunosuppressive function. Similarly, the analysis of exhausted CD8^+^ T cells and cytotoxic T cells revealed elevated levels of genes associated with dysfunction in TNBC, including CXCL13, CCL3, TIGIT, LAG3, HACVR2, and ENTPD1. Additionally, exhaustion-related transcription factors such as IFI16, IKZF3, ZNF683, PRDM1, and RBPJ were found to be present (46). The interaction of tumor cells with CD8^+^ T cells and myeloid cells in TNBC is of great significance as it facilitates the recruitment of CD8^+^ T cells and myeloid cells through the CX3CL1-CX3CR1 axis, thereby promoting immune infiltration (75, 104). Meanwhile, TNBC tumor cells induce the conversion of myeloid cells into suppressor cells by activating the JAG1-NOTCH2 signaling pathway and IL34-CSF1R axis (75, 105, 106). The presence of a substantial level of immune infiltration and immunosuppression is evident in TNBC (75).

#### 4.2.3 B lymphocyte-cell interactions

The involvement of B cells extends beyond antibody production as they actively modulate the tumor microenvironment through cytokine secretion and interactions with other immune cells. B cells exert regulatory effects on both tumor cells and immune cells. They modulate the activity and function of tumor cells, T lymphocytes, and macrophages via the secretion of cytokines like IL-10 and IL-6 (107, 108). Alternatively, B cells can present antigenic information to T lymphocytes, and in the context of interaction with CD4^+^ T lymphocytes, they facilitate the generation of antibodies (46). Moreover, B cells secrete cytokines, which drive the differentiation of T lymphocytes into memory T lymphocytes, thereby augmenting the establishment of long-term immune memory (52). An analysis of the interaction between T cells and B cells in TNBC revealed that the predominant interacting cell types were memory B cells, plasmablast cells, CTLA4-expressing T follicular helper (Tfh) cells, Tregs, and exhausted CD8 T cells (46). Moreover, memory B cells and Tfh cells in TNBC promote the positive cycle of plasma cell differentiation through the interaction between ICOSLG and ICOS, as well as the interaction between CXCL13 and CXCR5, thereby resulting in an increased proportion of plasma cells in TNBC (46). Direct interactions between B cells and tumor cells can be facilitated by ligand-receptor interactions. For instance, the binding of CD40 on the B cell surface to CD40L on the tumor cell surface promotes intercellular signal transduction (109). Moreover, the interaction between B cells and TNBC tumor cells, leading to an upregulation of the inflammatory cytokines IL-4 and IL-10 that drive the chronic inflammatory response of the tumor, may impede the antibody-mediated immune response (110). The regulation of B cell migration has been attributed to the release of chemokine CCL2 by TAMs. Furthermore, TAMs influence B cells through the expression of surface molecules, including CD40 and PD-L1, thereby impacting their proliferation and antibody secretion capacity (56, 111). These intricate cellular interactions maintain a suppressive immune microenvironment in TNBC, thereby impacting tumor proliferation and metastasis.

## 5 Discussion

TNBC is characterized by its heightened invasiveness, elevated lethality, unfavorable prognosis, and increased metastasis rate (112). The conventional treatment for TNBC primarily consists of surgery, radiotherapy, and chemotherapy. Given the absence of targeted therapy options, chemotherapy remains the primary approach for TNBC patients. Standard chemotherapeutic regimens encompass drugs such as paclitaxel, doxorubicin, and cyclophosphamide that effectively control tumor growth and metastasis (113). According to recent studies, neoadjuvant chemotherapy is frequently the primary treatment option for stage II or III TNBC, particularly when combined with immune checkpoint inhibitors like pembrolizumab, which may enhance treatment efficacy (114). Immunotherapy has gradually garnered attention in the management of TNBC (**Table 1**). Given tumor heterogeneity, clinical studies suggest that understanding the tumor microenvironment and cell-to-cell interactions can provide valuable guidance for treatment (9, 142–144). With the advancement of omics technology, the utilization of scRNA-seq and ST can facilitate the comprehensive characterization of the intricate tumor microenvironment in TNBC, thereby contributing significantly to the identification of biomarkers and therapeutic targets that hold importance in immunotherapy (145).

**Table 1:** Clinical trials of immunotherapy in TNBC.

Treatment	Clinical Trial Phase	ID	Reference
KN046 (diabody of PD-L1/CTLA-4) combination with nab-paclitaxel	phase Ib/II	NCT03872791	(115)
Cyclophosphamide, Pembrolizumab	Phase II	NCT02768701	(116)
AE37 peptide vaccination, Pembrolizumab	Phase II	NCT04024800	(117)
Multi-peptide—PVX-410 with Durvalumab	Phase I	NCT02826434	(118)
DNA—vaccinia p53MVA	Phase I	NCT02432963	(119)
Neo-antigen DNA, Durvalumab	Phase I	NCT03199040	(120)
MK-3475 (Pembrolizumab)	Phase I	NCT02977468	(121)
Pembrolizumab	Phase III	NCT02555657	(122)
Pembrolizumab, Paclitaxel (AlbuminBound)	Phase III	NCT02819518	(123)
Pembrolizumab And Carboplatin Plus Docetaxel	Phase II	NCT03639948	(124)
Atezolizumab (Anti PD-L1 Antibody)	Phase III	NCT03498716	(125)
Atezolizumab	Phase II	NCT03164993	(126)
Atezolizumab	Phase III	NCT03197935	(127)
multi-epitope folate receptor alpha peptide vaccine, sargramostim (GM-CSF), and cyclophosphamide	Phase II	NCT03012100	(128)
Folate Receptor Alpha (FRα) peptide vaccine mixed with GM-CSF as a vaccine adjuvant, with or without an immune priming with cyclophosphamide	Phase II	NCT02593227	(129)
Nab-paclitaxel, durvalumab, and tremelimumab with or without personalized synthetic long peptide vaccine (neoantigen vaccine)	Phase II	NCT03606967	(130)
mRNA c-Met-CAR-T	Early Phase I	NCT03060356	(131)
ROR1 CAR-specific Autologous T-Lymphocytes	Phase I	NCT02706392	(132)
THINK (THerapeutic Immunotherapy with NKR-2)	Phase I	NCT03018405	(133)
Autologous Tumor Infiltrating Lymphocytes (LN-145)	Phase II	NCT04111510	(134)
Oncolytic virus Talimogene Laherparepvec, Atezolizumab	Phase Ib	NCT03256344	(135)
LTX-315, Ipilimumab or Pembrolizumab	Phase I	NCT01986426	(136)
Pelareorep -/+ atezolizumab	Early Phase I	NCT04102618	(137)
Poliovirus (PVSRIPO)	Early Phase I	NCT03564782	(138)
Poxvirus (JX-594), Cyclophosphamide	Phase I/II	NCT02630368	(139)
Tavokinogene Telseplasmid (a plasmid encoding IL-12), pembrolizumab	Phase II	NCT03567720	(140)
Chemokine Modulation Therapy and Pembrolizumab	Early Phase I	NCT03599453	(141)

TNBC exhibits significant tumor heterogeneity, and scRNA-seq represents a potent tool for identifying distinct subtypes of this disease and elucidating their functional disparities. scRNA-seq has revealed four distinct subtypes of TNBC: IM-E, Str-E, DR-E, and Met-E. Among these subtypes, IM-E exhibits the most robust immunologic profile, the lowest level of intratumor heterogeneity, and the most favorable prognosis. In contrast, Met-E displays the highest degree of intratumor heterogeneity and is associated with the poorest prognosis (146). ScRNA-seq technology enables the analysis of gene temporal specificity and facilitates the acquisition of dynamic changes in cell type and gene expression (147). The pseudo-time trajectory of TNBC, for instance, initiates from HER2-negative tumor cells and subsequently diverges into two distinct branches, with cells in varying states exhibiting disparate biological characteristics (38). Cells exhibiting low HER2 expression demonstrate heightened metabolic activity and invasive characteristics, whereas the HER2-negative cells exhibit a distinct gene expression profile (38). The single-cell profiling of TNBC enables the classification of TNBC subtypes and reveals differences in tumor prognosis and cell composition among the different subtypes, providing valuable insights for personalized clinical treatment and prognosis.

The intricate tumor microenvironment and intercellular interactions also contribute to the pronounced heterogeneity of TNBC. TNBC demonstrates a notable immunosuppressive milieu, characterized by an enrichment of Treg cells, depletion of CD8^+^ T cells, and heightened presence of plasma cells (46). The immunosuppressive microenvironment in breast cancer is constructed through interactions between immunodeficient cell populations, such as Tregs and CAFs, and other immune cells, indicating their limited tumor spatial specificity (75). The spatial distribution of these cells has been analyzed, with Treg cells typically found in proximity to effector T cells, resulting in the suppression of immune responses within these regions (101, 102). Interaction analysis of T cells and B cells also revealed that the reciprocal activation of memory B cells and Tfh cells facilitates a positive feedback loop in plasma cell differentiation through the ICOSLG-ICOS interaction and CXCL13-CXCR5 interaction, ultimately resulting in an augmented proportion of plasma cells (46). The scRNA-seq and ST techniques enable comprehensive investigations of cells and their intercellular interactions in tumors, surpassing the capabilities offered by traditional immunohistochemistry and other methodologies (148). The integration of scRNA-seq and ST technology expands the scope of single-cell analysis to encompass investigations into intercellular interactions, thereby enhancing our understanding of cellular plasticity and tumor immunology and prognosis (149–151). The remaining challenges include the need for improved spatial resolution (152). Different spatially resolved high-throughput imaging technology platforms, such as cyclic immunofluorescence (Cyclic IF), imaging mass cytometry (IMC), and multiplex ion beam imaging (MIBI), play a crucial role in validating biomarkers and bridging the gap between molecular subtypes and clinical applications. These technologies can identify biomarkers with prognostic value at the single-cell level, precisely define tumor subtypes with prognostic significance, clearly explain the internal relationships between different molecular subtypes and clinical characteristics and strongly promote the innovative development of personalized treatment for TNBC (153, 154).

In addition, certain technical errors may occur during the acquisition of single-cell data. For example, the isolation of single cells may not be completely accurate, and the data may be mixed with information from a small number of other impurity cells, which affects the precision of cell subtype classification. When analyzing the data from scRNA-seq and ST, due to limitations of algorithms, there may be insufficient exploration of the potential information in the data, and it is easy to overlook the capture of low-abundance transcripts or weak interaction signals between cells. Moreover, when integrating the technologies of scRNA-seq and ST, problems such as inconsistent data formats and difficulties in matching spatial resolution with single-cell resolution also arise.

In the future, to achieve more personalized and precise treatment, it is essential to analyze the cellular transcriptome, protein expression, and cell metabolism. Combining scRNA-seq and ST with spatial proteomics/metabolomics can provide therapeutic targets for TNBC, enabling more accurate and personalized treatment. The biomarkers identified by scRNA-seq and ST technologies can help diagnose the subtypes to which patients belong more accurately in the early stage of the disease and formulate more targeted treatment plans. Moreover, a deeper understanding of the tumor microenvironment and the interactions between cells can facilitate the development of new therapeutic drugs targeting corresponding molecules or combined treatment strategies, thereby improving the cure rate of TNBC.

## 6 Conclusion

In summary, this review provides an overview of scRNA-seq and ST technologies, elucidating their role in characterizing cell subsets and analyzing cell-cell interactions within TNBC tumor tissues. In doing so, it offers insights into pathogenesis, tumor progression and metastasis, drug development, and treatment strategies for TNBC.
